# Vegetarian diet-induced increase in linoleic acid in serum phospholipids is associated with improved insulin sensitivity in subjects with type 2 diabetes

**DOI:** 10.1038/nutd.2013.12

**Published:** 2013-06-17

**Authors:** H Kahleova, M Matoulek, M Bratova, H Malinska, L Kazdova, M Hill, T Pelikanova

**Affiliations:** 1 Diabetes Centre, Institute for Clinical and Experimental Medicine, Prague, Czech Republic; 2 Charles University, First Faculty of Medicine, Prague, Czech Republic; 3 Institute of Endocrinology, Prague, Czech Republic

**Keywords:** fatty acid composition of serum phospholipids, insulin resistance, linoleic acid, type 2 diabetes, vegetarian diet, visceral fat

## Abstract

**BACKGROUND AND AIMS:**

Fatty acids are important cellular constituents that may affect many metabolic processes relevant for the development of diabetes and its complications. We showed previously that vegetarian diet leads to greater increase in metabolic clearance rate of glucose (MCR) than conventional hypocaloric diet. The aim of this secondary analysis was to explore the role of changes in fatty acid composition of serum phospholipids in diet- and exercise-induced changes in MCR in subjects with type 2 diabetes (T2D).

**METHODS:**

Subjects with T2D (*n*=74) were randomly assigned into a vegetarian group (VG, *n*=37) following vegetarian diet or a control group (CG, *n*=37) following a conventional diet. Both diets were calorie restricted (−500 kcal day^–1^). Participants were examined at baseline, 12 weeks of diet intervention and 24 weeks (subsequent 12 weeks of diet were combined with aerobic exercise). The fatty acid composition of serum phospholipids was measured by gas liquid chromatography. MCR was measured by hyperinsulinemic isoglycemic clamp. Visceral fat (VF) was measured by magnetic resonance imaging.

**RESULTS:**

Linoleic acid (LA; 18:2n6) increased in VG (*P*=0.04), whereas it decreased in CG (*P*=0.04) in response to dietary interventions. It did not change significantly after the addition of exercise in either group (group × time *P*<0.001). In VG, changes in 18:2n6 correlated positively with changes in MCR (*r*=+0.22; *P*=0.04) and negatively with changes in VF (*r*=−0.43; *P*=0.01). After adjustment for changes in body mass index, the association between 18:2n6 and MCR was no longer significant. The addition of exercise resulted in greater changes of phospholipid fatty acids composition in VG than in CG.

**CONCLUSION:**

We demonstrated that the insulin-sensitizing effect of a vegetarian diet might be related to the increased proportion of LA in serum phospholipids.

## Introduction

The fatty acid composition of membrane phospholipids may affect the biophysical properties of cell membranes modulating both insulin binding and action.^1^ Diet-induced alterations in membrane composition may provide a mechanism for improving the cellular response to insulin.^2, 3^

The fatty acid composition in serum phospholipids is routinely measured in humans and reflects both the effects of dietary fatty acid intake and endogenous fatty acid metabolism including their synthesis, β-oxidation, de-saturation, elongation and lipoperoxidation.^4^

It has been shown that fatty acid composition of both serum phospholipids and adipose tissue lipids are valid biomarkers of dietary intake in men.^1, 5^ The fatty acid composition in serum phospholipids correlates with the composition of tissue lipids.^1^ The content of polyunsaturated fatty acids of the diet has been shown to correlate with the fatty acid composition in adipose tissue and all blood lipid fractions.^6^ Correlations are weaker for monounsaturated and saturated fatty acids.^1^ We have previously demonstrated a negative correlation between the content of saturated fatty acids in plasma phospholipids and insulin sensitivity and a positive association between insulin action and the proportion of linoleic acid (LA) in healthy individuals.^6^

Serum fatty acid composition predicts the long-term development of the metabolic syndrome.^7^ Insulin resistance and insulin-resistant states are often associated with the serum fatty acid pattern, characterized by an increased proportion of palmitic and a low proportion of LAs, with a distribution of other fatty acids indicating an increased activity of Δ9 and Δ6 desaturase and decreased Δ5 desaturase activity.^8^ This corroborates the concept that there may be a causal relationship between the type of fat in the diet and insulin action.^2^

Observational studies have indicated that dietary fat quality may be related to the development of insulin resistance and the metabolic syndrome, also independent of possible effects on body weight.^2^ Intervention studies have shown that the plasma fatty acid pattern changes after substituting a monounsaturated fatty acid diet for a saturated fatty acid diet, resulting in increased insulin sensitivity.^9^

A vegetarian diet has been shown to be an efficient tool to provide weight loss and improve glycemic control compared with a conventional diabetic diet.^10^ As we showed previously, a vegetarian diet leads more effectively to an increase in insulin sensitivity, reduction in visceral fat (VF) and improvement in oxidative stress markers than a conventional diabetic diet in subjects with type 2 diabetes (T2D).^11^ Insulin resistance has been associated with the accumulation of fat within skeletal muscle fibers as intramyocellular lipid.^12, 13^ Vegetarians have lower intramyocellular lipid concentrations than non-vegetarians.^14^ As to whether a vegetarian diet might alter the composition of serum phospholipids, and whether these changes are associated with insulin sensitivity remains, to the best of our knowledge, to be clarified.

Besides dietary factors, exercise has been shown as an important factor in both prevention and treatment of T2D because of its insulin-sensitizing effect.^15, 16^ It has been shown that long term, intense physical training significantly affects the fatty acid composition of membrane phospholipids.^17^ To our best knowledge, the effect of a vegetarian diet compared with a conventional diet on the fatty acid composition of membrane phospholipids after the aerobic exercise program has not been studied yet.

The aim of this secondary analysis of a previously published data was to explore the mechanisms of the insulin-sensitizing effect of vegetarian diet in T2D. Our hypothesis was that increased insulin sensitivity induced by a vegetarian diet and aerobic exercise would be related to changes in the serum phospholipids fatty acid pattern.

## Subjects and methods

The characteristics of the sample and the methods are described in detail elsewhere.^1^ Briefly:

### Subjects

Seventy-four subjects with T2D treated by oral hypoglycemic agents, both men (47%) and women (53%), age 30 to 70 years, glycated hemoglobin between 6 and 11% (42 to 97 mmol mol^–1^), body mass index (BMI) between 25 and 53 kg m^−2^, were recruited for the intervention program.

### Study design

A 24 weeks, randomized, open, parallel design was used. The subjects were randomly assigned to either into the vegetarian group (VG, *n*=37) who received a vegetarian diet or the control group (CG, *n*=37) who received a conventional diabetic diet. Both diets were designed to be isocaloric and calorie restricted (−500 kcal day^–1^) with caloric intakes based on the measurement of resting energy expenditure of each subject by indirect calorimetry (metabolic monitor VMAX; Sensor Medics, Anaheim, CA, USA).^18^ The second 12 weeks of the diet were combined with aerobic exercise. All meals during the study were provided. Participants were examined at baseline, 12 and 24 weeks. The study protocol was approved by the Institutional Ethics Committee.

### Diet

The vegetarian diet (∼60% of energy from carbohydrates, 15% protein and 25% fat) consisted of vegetables, grains, legumes, fruits and nuts. Animal products were limited to maximum of one portion of low-fat yogurt a day. The conventional diabetic diet was administered according to the dietary guidelines of the Diabetes and Nutrition Study Group of the European Association for the Study of Diabetes. It contained 50% of total energy from carbohydrates, 20% protein, <30% fat (⩽7% saturated fat, <200 mg day^–1^ of cholesterol per day).

### Compliance

Records of all visits to pick up meals were kept. At weeks 0, 12 and 24, a 3-day dietary record was completed by each participant (2 weekdays and 1 weekend day). A registered dietician analyzed all 3-day dietary records using country-specific food-nutrient database.^19^ At weeks 3, 8, 14 and 19, a registered dietician made unannounced telephone calls and each participant recalled his or her 24-h diet. This data set was not statistically analyzed, but allowed the investigators to check the adherence and to provide additional counseling.

### Exercise program

Participants were asked not to alter their exercise habits during the first 12 weeks. During weeks 13–24, they were prescribed an individualized exercise program based on the history of physical activity and initial spiroergometric examination. Heart rate for training has been prescribed according to Borg's Scale Rate of perceived exertion^11, 12, 13, 14^ and measurement of heart rate during spiroergometry. Participants exercised at 60% of maximal heart rate twice a week for 1 h under professional supervision plus once a week at home or at the sports center with the same intensity; they were given a sport-tester and a pedometer for individual physical activities and were repeatedly instructed on how to use them. Physical activity was assessed by pedometer Omron HJ-113 (Omron, Kyoto, Japan): each participant completed a 3-day record, 2 weekdays and 1 weekend day, and with two questionnaires: the International Physical Activity Questionnaire^20^ and the Baecke questionnaire^21^ at weeks 0, 12 and 24. Records of each participant's visits at the sports center were kept.

### Medication

Participants were asked to continue their pre-existing medication regimens, except when hypoglycemia occurred repeatedly (plasma glucose determined at the laboratory <4.4 mmol l^−1^ or capillary glucose reading <3.4 mmol l^−1^ accompanied by hypoglycemic symptoms). In such cases, medications were reduced by a study physician following the medication protocol. All participants were given an Accu-Chek Go glucometer (Roche, Basel, Switzerland) and were instructed on how to use it.

### Procedures

All measurements were performed at 0, 12 and 24 weeks on an outpatient basis, after 10–12 h overnight fasting with only tap water allowed *ad libitum*.

#### Hyperinsulinemic isoglycemic clamp

To measure insulin sensitivity, the hyperinsulinemic (1 mU kg^−1^ min^−1^) isoglycemic clamp, lasting 3 h, was conducted as previously described.^6, 22^ Insulin sensitivity was estimated as the metabolic clearance rate of glucose (MCR) calculated during the last 20 min of the clamp after correction for changes in glucose pool size.^6, 22^

#### Magnetic resonance imaging

To measure the volume of visceral and subcutaneous fat, magnetic resonance imaging of the abdomen was performed. Twenty-seven water-suppressed magnetic resonance images centered to the intervertebral disc of L2/L3 with repetition time/echo time=450/10 ms and thickness of 10 mm were acquired in breath-hold. The post-processing of magnetic resonance imaging with the calculation of subcutaneous and visceral abdominal fat volume was done in MATLAB (The Math Works, Natick, MA, USA); the inner border of subcutaneous region was detected semi-automatically,^23^ while the abdominal fat voxels were selected by thresholding.

### Analytic methods

#### Phospholipid fatty acid composition

Serum total lipids were extracted by chloroform:methanol (2:1) according to a modified Folch method.^24^ Phospholipids were isolated by thin layer chromatography using hexane–diethylether–acetic acid (80:20:3, v/v) as a solvent system. Fatty acid in serum phospholipids was converted to methyl esters using 1% solution of Na in methanol and the fatty acid methyl esters were eluted with hexane. Gas chromatography of the fatty acid methyl esters was performed on a GS 5890A (Hewlett Packard, Palo Alto, CA, USA) instrument equipped with a flame-ionization detector. A carbowax-fused silica capillary column (25 m × 0.25 mm i.d.) was used. The column temperature was 150, 225 °C (2 °C min^–1^), hydrogen was used as the carrier gas.^25^ Individual peaks of fatty acid methyl esters were identified by comparing retention times with those of authentic standards (Sigma, Prague, Czech Republic). The composition of serum fatty acid (spectrum of 17 main fatty acid) was analyzed. The product/precursor ratios of the serum fatty acid were used to calculate indices reflecting the activities of enzymes involved in fatty acid metabolism:elongase (18:0/16:0), D6 desaturase (18:3n8/18:2n6), D5 desaturase (20:4n6/20:3n6 and D9 desaturase (16:1n7/16:0).^6, 22^

Plasma lipids concentrations were measured by enzymatic methods (Roche). High-density lipoprotein cholesterol was measured after double precipitation with dextran and MgCl_2_. Low-density lipoprotein cholesterol was estimated using the Friedewald equation, if triglyceride concentration was within normal limits.

### Statistical analyses

The intention-to-treat analysis included all participants. A repeated-measures analysis of variance models with between-subject and within-subject factors and interactions were used for evaluation of the relationships between continuous variables and factors. Factors group, subject and time were included in the model. Interactions between group and time (group × time) were calculated for each variable. Within each group, paired comparison *t*-tests were calculated to test whether the changes from baseline to 12 weeks and from 12 to 24 weeks were statistically significant. Pearson correlations were calculated for the relationship between changes in each individual fatty acid of serum phospholipids and changes in MCR and volume of VF. Regression analysis was used to adjust the correlation for changes in BMI.

## Results

In all, 92% of the participants completed the first 12 weeks (95% in VG and 89% in CG); 84% of the participants in each group completed all 24 weeks. Adherence to the prescribed diet at 24 weeks was 72.5% in VG and 71% in CG. Pedometer readings and self-reported energy expenditure showed no significant between-group differences. Adherence to the prescribed exercise program was 85.5% (90.3% in VG and 80.6% in CG).

### Dietary intake

Parameters of dietary intake are shown in Table 1. Both groups reduced energy intake at the beginning of the study (weeks 0–12) and the reduced energy intake remained also in subsequent 12 weeks during the exercise program (weeks 12–24). The percentage of consumed calories in the form of carbohydrates increased significantly in VG (weeks 0–12), whereas it did not change in CG. The percentage of consumed calories in the form of fats decreased in VG, whereas the decrease in CG was nonsignificant. The percentage of consumed calories in the form of proteins decreased significantly in VG, whereas it did not change in CG. Cholesterol intake decreased significantly only in VG.

### Body weight and VF

Body weight and volume of VF are shown in Table 1. Body weight decreased in both groups in response to the dietary interventions, more in VG. It was maintained after the addition of exercise in both groups. The volume of VF decreased in both groups after the dietary interventions, more in VG. After the addition of exercise, it further decreased in VG, whereas it remained unchanged in CG. The results are discussed extensively elsewhere.^1^

### Glycemic control and insulin sensitivity

Glycated hemoglobin decreased in both groups in response to the dietary interventions and did not change significantly after the addition of exercise. The MCR increased in both groups during the first 12 weeks. After the addition of exercise, there were nonsignificant trends for an increase in VG and a decrease in CG. For more details, we refer to Table 1 and to our previous work.^1^

### Plasma lipids

Neither total cholesterol nor triglycerides did change in either group in either period. Low-density lipoprotein cholesterol decreased in VD compared to CG (weeks 12–0). High-density lipoprotein cholesterol increased in VG in response to exercise (weeks 24-12), while in CG it increased only from baseline to 24 weeks. Free fatty acids did not change in either group in either period. They increased in CG by 41% from weeks 0 to 24 (*P*=0.02; group × time *P*=0.2). Plasma lipids are shown in Table 1 and are discussed more extensively elsewhere.^1^

#### Fatty acid composition in serum phospholipids

Relative contents of all measured fatty acids in serum phospholipids at weeks 0, 12 and 24 in both groups are shown in Table 2.

**Figure 1:**
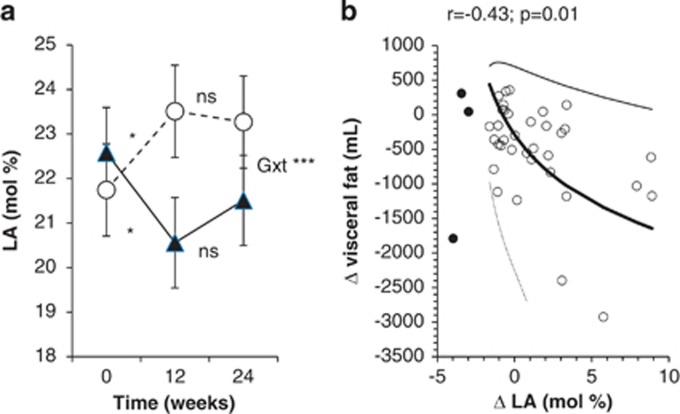
(**a**) The content of LA (18:2n6) in serum phospholipids at start, 12 and 24 weeks. The CG: triangles, full line; the VG: circles, dashed line. Error bars represent 95% confidence intervals (CIs). *P*-values: *P<0.05, ***P<0.001. Gxt: *P*-value for interaction between factors group and time (analysis of variance). (**b**) Correlation between changes in LA (18:2n6) and changes in volume of VF in VG. Full line: the main correlation axis, dotted line: the 95% confidence ellipsoid. NS, not significant.

*N6 polyunsaturated fatty acids* did not change in either group in response to dietary interventions. They decreased after the addition of exercise training in VG (*P*<0.001), whereas the trend toward decrease in CG was insignificant. There were no significant differences in the total n6 polyunsaturated fatty acids between the groups. Patients in the VG exhibited increased content of LA by 10% compared with the CG (group × time *P*<0.001; Figure 1a). Arachidonic acid decreased after the addition of exercise in VG, whereas it did not change significantly in CG.

*N3 polyunsaturated fatty acids* did not change in VG in response to dietary intervention, whereas there was a insignificant trend toward an increase in CG. After the addition of exercise, they decreased in VG (*P*<0.001), whereas the trend toward a decrease in CG was insignificant.

*Monounsaturated fatty acids* did not change in either group in either period. Palmitooleic acid increased in both groups in response to exercise.

*Saturated fatty acids* did not change in either group in response to dietary interventions. After the addition of exercise, both groups exhibited significantly increased content of saturated fatty acids, mainly the palmitic acid, whereas stearic acid decreased in the VG after the addition of exercise.

The ratio of saturated to unsaturated fatty acids did not change in either group in response to dietary interventions. After the addition of exercise it increased in VG (*P*<0.001), whereas the increase in CG was not significant.

### The enzymes

There were no significant changes in fatty acid enzyme activity in response to dietary intervention in either group. However, after the addition of exercise, decreased activity of elongase (*P*<0.01) and increased activity of Δ9 desaturase (*P*=0.003) were observed in both groups.

### Correlations

In VG, changes in the LA correlated positively with changes in metabolic clearance rate of glucose (*r*=+0.22; *P*=0.04) and negatively with changes in volume of visceral fat (*r*=−0.43; *P*=0.01; Figure 1b). After adjustment for changes in BMI, the association between LA and MCR was no longer significant. Furthermore, changes in the LA correlated positively with changes in high-density lipoprotein cholesterol (*r*=+0.36; *P*=0.01). The correlation between changes in the LA and neither triglycerides, total cholesterol nor low-density lipoprotein cholesterol was significant (*P*=0.07; *P*=0.08 and *P*=0.27, respectively).

In CG, changes in the docosapentaenoic acid correlated positively with changes in MCR (*r*=+0.2; *P*=0.05) and negatively with changes in volume of VF (*r*=−0.36; *P*=0.03).

## Discussion

We demonstrated that vegetarian diet increases the content of LA in serum phospholipids. Increased content of LA was associated with increased insulin sensitivity in VG. This result is in accordance with previous research showing the beneficial effects of increased content of LA in serum phospholipids on insulin action.^6, 26^ It suggests that increased content of LA may be a potential mechanism of the insulin-sensitizing effect of a vegetarian diet.

According to the metabolomic research, linoleic and palmitic acids belong to the metabolites, which were identified as potential biomarkers for diabetes mellitus.^27^ In a cohort of middle-aged normoglycemic men (*n*=895) in a Finnish prospective cohort study with follow-up after 4 years, men with a high proportion of LA in plasma fatty acids, indicating a high intake of dietary LA, had a lower risk of developing diabetes and showed lower increases in serum insulin and blood glucose over the follow-up period. This is comparable with earlier findings^28^ and is also in line with dietary epidemiology,^29^ which indicated that individuals with a low proportion of LA or vegetable fat in the diet have an increased risk of developing T2D.

Content of LA in plasma lipids has also been directly related to other features of the metabolic syndrome, particularly plasma lipid concentrations and blood pressure. The increase in the proportion of LA in serum phospholipids corresponded with decreases in serum cholesterol^30^ and was inversely related to the incidence of hypertension.^31^ Low proportion of LA predicted the development of left ventricular hypertrophy.^32^ Furthermore, the content of LA was positively related to an endothelial function index. Endothelial dysfunction may represent a possible link between diet, fatty acid profile in plasma, sustained hypertension and left ventricular hypertrophy.^2^ In our study, we confirmed a positive association between changes in the content of LA in serum phospholipids and plasma high-density lipoprotein cholesterol.

After adjustment for changes in BMI the association between LA and insulin sensitivity was no longer significant. The effect of increased content of LA on insulin sensitivity may be directly linked to specific effects of vegetarian diet and the loss of association after the adjustment for changes in BMI could be explained by a small number of study subjects. But it is also possible that the increase in the content of LA may be directly related to changes in BMI (because BMI decreased more in VG compared with CG). Also, there is potential for other factors to confound this association (dyslipidemia, oxidative stress, inflammation, endothelial dysfunction, and so on).

In CG, changes in insulin sensitivity and VF were associated with changes in docosapentaenoic acid. An increase in the content of the docosapentaenoic acid in muscle lipids after very low calorie diet was described previously.^33^ Docosapentaenoic acid was the only long-chain polyunsaturated n3 structural fatty acid that correlated with insulin sensitivity in patients with coronary artery disease.^34^ It cannot be ruled out, therefore, that docosapentaenoic acid may have a role in insulin action in the present setting. However, caution is needed in drawing clear-cut conclusions because docosapentaenoic acid is found in small amounts in serum phospholipids.

There was no significant change in either saturated, monounsaturated, n3- or n6-polyunsaturated fatty acids as a whole in response to dietary intervention. In this regard, there was no significant difference between the groups.

Another factor we studied was the addition of physical exercise. The decrease in the content of arachidonic acid in VG after the addition of exercise is remarkable. As arachidonic acid is the precursor of the proinflammatory prostaglandins, leukotriens, thromboxane A2 and prostacyclin, its decrease may have beneficial effects on inflammation, thrombogenesis and vasoconstriction.

The increase in saturated fatty acids in both groups during the exercise training, namely myristic and palmitic acid, is in accordance with some experimental studies showing increase in the content of saturated fatty acids in the membrane phospholipids in response to physical exercise, which has been explained by the increase of the membrane stability and reduced lipoperoxidation.^35^ Palmitic acid is the initial major product of lipid synthesis *de novo* from acetyl-CoA. Palmitic acid can be further converted by Δ9 desaturation to palmitooleic acid.^36^

The limitations of our study are that 16% of the patients did not complete the study. We also measured all fatty acids only as proportions of total fatty acids.

In conclusion, we showed that vegetarian diet increased the content of LA in serum phospholipids and its changes were associated with changes in insulin sensitivity and VF. The results support the hypothesis that the insulin-sensitizing effect of vegetarian diet may be mediated by changes in fatty acid pattern in serum phospholipids in subjects with T2D. The addition of exercise resulted in greater changes of phospholipid fatty acids composition in VG than in CG. This is in accordance with previous consensus that the changes in fatty acid composition may have a role in the modulation of insulin action in peripheral tissues.

## Figures and Tables

**Table 1:** Dietary intake, anthropometric and laboratory parameters

	*Vegetarian group (*n*=37)*			*Control group (*n*=37)*			P*-value*
	*Baseline*	*12 Weeks*	*24 Weeks*	*Baseline*	*12 Weeks*	*24 Weeks*	
Energy intake (kcal)	1835 (1623–1906)	1718 (1563–1872)***	1636 (1295–1711)	1832 (1705–1968)	1659 (1575–1853)***	1694 (1468–1960)	0.120
Carbohydrates (% of kcal)	42 (39–44)	52 (48–54)**	54 (50–59)	42 (41–44)	42 (40–44)	46 (43–50)	0.080
Fat (% of kcal)	38 (36.6–40)	35 (33–36.4)*	32 (31–36)	38 (35–40)	36 (34–38)	35 (31–39)	0.610
Proteins (% of kcal)	20.6 (19.3–21.5)	15 (14–16)***	16 (15–17)	18.4 (17.3–19.2)	19 (18–20)	18 (16–20)	<0.001
Cholesterol intake (mg day^–1^)	368 (315–424)	28 (14–47)***	53 (38–86)	348 (297–405)	296 (250–350)	287 (210–376)	<0.001
P/S ratio	0.69 (0.66–0.71)	1.39 (1.28–1.53)***	1.13 (1.0–1.3)	0.53 (0.44–0.61)	0.7 (0.66–0.75)*	0.62 (0.57–0.68)	0.01
Saturated fatty acids (g day^–1^)	24.9 (22.4–27.7)	18.0 (15.9–20.4)	13.9 (11.4–17.0)	29.3 (26.4–23.6)	23.4 (21.0–26.0)	22.7 (18.8–27.4)	0.37
Monounsaturated fatty acids (g day^–1^)	21.9 (19.5–24.6)	21.5 (18.9–24.6)	14.4 (11.8–17.6)	24.1 (21.4–27.1)	21.3 (19.0–24.0)	21.2 (17.3–26.0)	0.26
Polyunsaturated fatty acids (g day^–1^)	15.4 (14.0–17.2)	20.8 (18.1–24.4)	15.2 (12.8–18.5)	14.6 (13.2–16.4)	14.7 (13.3–16.4)	13.9 (11.8–16.7)	0.24
Fiber intake (g day^–1^)	23 (20–25)	29 (26–32)***	28 (26–30)	23 (21–26)	21.5 (20.5–22.3)	23 (21–25)	0.030
Weight (kg)	101.1 (100.5–101.8)	95.2 (94.5–95.8)***	95.2 (94.5–95.9)	100.8 (100.1–101.5)	97.5 (96.8–98.3)***	98.0 (97.3–98.7)	<0.001
BMI (kg m^–2^)	35.1 (34.9–35.3)	33.1 (32.9–33.3)***	33.2 (33.0–33.4)	35.0 (34.7–35.2)	34.0 (33.8–34.3)	34.1 (33.9–34.3)	<0.001
Volume of visceral fat (ml)	4266 (4183–4350)	3896 (3852–3963)***	3811 (3723–3848)*	4275 (4189–4380)	4132 (4048–4180)*	4169 (4078–4260)	0.007
MCR (ml kg^–1^ min^–1^)	2.3 (2.1–2.5)	2.9 (2.7–3.2)***	3.0 (2.8–3.2)	2.2 (1.9–2.4)	2.9 (2.7–3.1)***	2.7 (2.5–2.9)	0.04
HbA1c (mmol mol^–1^, IFCC)	60.0 (57.8–63.2)	54.1 (52.5–55.9)***	54.5 (52.5–56.6)	61.0 (59.1.0–62.7)	56.3 (55.1–57.6)***	58.3 (57.0–59.7)	0.370
Total cholesterol (mmol l^–1^)	4.4 (4.28–4.53)	4.3 (4.17–4.42)	4.3 (4.18–4.43)	4.2 (4.07–4.33)	4.1 (3.96–4.23)	4.1 (3.97–4.24)	0.340
HDL-cholesterol (mmol l^–1^)	1.07 (1.00–1.09)	1.02 (0.98–1.06)	1.11 (1.06–1.15)*	1.09 (1.02–1.12)	1.11 (1.06–1.15)	1.13 (1.09–1.17)	0.070
LDL-cholesterol (mmol l^–1^)	2.54 (2.40–2.68)	2.22 (2.10–2.38)*	2.25 (2.12–2.38)	2.57 (2.41–2.73)	2.48 (2.33–2.64)	2.56 (2.41–2.72)	0.05
Free fatty acids (mmol l^–1^)	0.54 (0.44–0.64)	0.44 (0.36–0.54)	0.59 (0.48–0.72)	0.58 (0.48–0.72)	0.70 (0.56–0.86)	0.78 (0.65–0.92)	0.200
Triglycerides (mmol l^–1^)	2.12 (1.98–2.25)	2.03 (1.88–2.17)	1.91 (1.78–2.04)	2.10 (1.96–2.24)	1.95 (1.81–2.08)	2.05 (1.91–2.18)	0.560

Abbreviations: ANOVA, analysis of variance; BMI, body mass index; HbA1c, glycated hemoglobin; HDL, high-density lipoprotein; IFCC, The International Federation of Clinical Chemistry and Laboratory Medicine; LDL, low-density lipoprotein; MCR, metabolic clearance rate of glucose; P/S ratio, ratio of polyunsaturated to saturated fatty acids.

Data are means±95% confidence intervals. Listed *P*-values are from ANOVA for interaction between group (vegetarian and control group) and time (0,12 and 24 weeks). Significant changes from baseline to 12 weeks and from 12 to 24 weeks are indicated by * for *P*<0.05, ** for *P*<0.01 and *** for *P*<0.001.

**Table 2:** Fatty acids in serum phospholipids (mol %)

*Fatty acids in serum phospholipids (mol %)*	*Vegetarian group (*n*=37)*			*Control group (*n*=37)*			P*-value*
	*Baseline*	*12 Weeks*	*24 Weeks*	*Baseline*	*12 Weeks*	*24 Weeks*	
*Saturated fatty acids*	33.5 (30.9–35.9)	32.6 (30.0–35.1)	41.2 (38.9–43.4)***	32.6 (30.0–35.0)	33.5 (31.0–35.9)	36.2 (33.8–38.5)	0.037
Myristic acid (14:00)	0.01 (0.006–0.02)	0.01 (0.006–0.02)	0.13 (0.08–0.225)***	0.01 (0.005–0.02)	0.01 (0.004–0.02)	0.04 (0.025–0.07)*	0.110
Palmitic acid (16:00)	16.2 (13.4–18.9)	15.3 (12.5–18.0)	26.5 (23.7–29.3)***	15.6 (12.8–18.3)	14.1 (11.4–16.8)	20.5 (17.8–23.3)***	0.111
Stearic acid (18:00)	16.5 (15.9–17.1)	16.3 (15.7–17.0)	14.3 (13.8–14.8)***	16.4 (15.8–17.0)	15.8 (15.2–16.5)	15.1 (14.5–15.7)	0.061
Arachidic acid (20:00)	0.16 (0.11–0.25)	0.21 (0.14–0.33)	0.13 (0.08–0.19)	0.17 (0.11–0.26)	0.21 (0.14–0.31)	0.13 (0.09–0.2)	0.978
*Monounsaturated fatty acids*	12.2 (11.6–12.8)	12.7 (12.1–13.4)	12.4 (11.8–13.0)	12.2 (11.7–12.8)	12.3 (11.7–12.9)	12.5 (12.0–13.2)	0.551
Palmitooleic acid (16:1n7)	0.16 (0.11–0.21)	0.15 (0.10–0.21)	0.5 (0.36–0.60)***	0.13 (0.09–0.18)	0.10 (0.06–0.14)	0.22 (0.16–0.29)***	0.081
Oleic acid (18:1n9)	10.3 (9.8–10.8)	10.3 (9.8–10.8)	10.1 (9.7–10.7)	9.9 (9.4–10.3)	10.0 (9.5–10.5)	10.4 (10.0–11.0)	0.304
Vaccenic acid (18:1n7)	1.4 (1.3–1.5)	1.6 (1.5–1.7)	1.4 (1.3–1.5)	1.7 (1.5–1.8)	1.7 (1.5–1.8)	1.7 (1.5–1.8)	0.216
Gondoic acid (20:1n9)	0.16 (0.11–0.21)	0.47 (0.34–0.64)***	0.06 (0.05–0.09)***	0.30 (0.22–0.41)	0.44 (0.32–0.60)	0.14 (0.11–0.20)***	0.016
*N3 polyunsaturated fatty acids*	14.1 (13.1–15.2)	14.0 (13.1–15.1)	11.7 (11.1–12.4)***	13.6 (12.8–14.6)	15.2 (14.1–16.5)	13.3 (12.5–14.2)	0.034
α-Linolenic acid (18:3n3)	0.22 (0.12–0.31)	0.15 (0.05–0.24)	0.1 (0.05–0.2)	0.15 (0.05–0.26)	0.13 (0.04–0.23)	0.14 (0.03–0.23)	0.256
Eicosapentaenoic acid (20:5n3)	1.0 (0.7–1.3)	0.8 (0.5–1.0)	1.1 (0.8–1.4)	0.6 (0.4–0.8)	1.2 (0.9–1.5)**	1.5 (1.2–1.9)	0.002
Docosapentaenic acid (22:5n3)	1.6 (1.3–1.9)	1.6 (1.3–1.9)	1.5 (1.3–1.8)	1.7 (1.4–2.0)	2.3 (1.9–2.9)*	1.5 (1.3–1.8)**	0.121
Docosahexaenoic acid (22:6n3)	6.0 (4.9–7.2)	5.6 (4.5–6.8)	2.3 (1.7–3.1)***	5.1 (4.1–6.2)	4.7 (3.7–5.7)	4.6 (3.7–5.7)	<0.001
*N6 polyunsaturated fatty acids*	50.9 (49.3–52.6)	52.3 (50.6–54.2)	46.1 (44.7–47.6)***	52.6 (51.0–54.4)	51.7 (50.0–53.5)	49.3 (47.8–50.8)	0.059
Linoleic acid (18:2n6)	21.7 (20.7–22.5)*	23.5 (22.5–24.5)	23.3 (22.2–24.3)	22.6 (21.6–23.6)	20.6 (19.5–21.6)*	21.5 (20.5–22.5)	<0.001
γ-Linolenic acid (18:3n6)	0.2 (0.17–0.25)	0.2 (0.17–0.24)	0.2 (0.17–0.24)	0.15 (0.12–0.18)	0.09 (0.07–0.12)*	0.15 (0.12–0.18)	0.067
Eicosadienoic acid (20:2n6)	0.61 (0.54–0.69)	0.71 (0.62–0.81)	0.52 (0.46–0.59)**	0.64 (0.56–0.72)	0.77 (0.68–0.88)	0.58 (0.51–0.66)**	0.889
Dihomo-γ-linolenic acid (20:3n6)	5.3 (4.8–5.9)	5.4 (4.8–6.0)	4.3 (4.0–4.8)**	5.1 (4.7–5.7)	4.7 (4.3–5.2)	4.7 (4.3–5.2)	0.131
Arachidonic acid (20:4n6)	16.3 (15.0–17.6)	15.5 (14.2–16.8)	11.5 (10.4–12.6)***	17.6 (16.3–18.9)	17.9 (16.5–19.3)	15.9 (14.7–17.2)	0.027
Eicosatetraenoic acid (22:4 n6)	0.3 (0.25–0.36)	0.27 (0.22–0.32)	0.18 (0.15–0.22)**	0.26 (0.22–0.31)	0.26 (0.22–0.31)	0.25 (0.21–0.3)	0.036
The ratio of n6 to n3	11.5 (11.0–12.1)	11.7 (11.1–12.3)	13.0 (12.4–13.7)***	11.9 (11.3–12.5)	11.0 (10.5–11.6)	11.9 (11.4–12.6)	0.067
*The ratio of saturated to unsaturated FA*	0.51 (0.46–0.56)	0.49 (0.43–0.54)	0.7 (0.65–0.76)***	0.49 (0.44–0.54)	0.47 (0.41–0.52)	0.57 (0.52–0.62)	0.056
Elongase (the ratio of 18:00 to 16:00)	7.0 (6.8–7.3)	7.1 (6.9–7.4)	6.5 (6.4–6.6)**	7.1 (6.9–7.3)	7.2 (7.0–7.6)	6.7 (6.6–6.9)**	0.12
Δ6 desaturase (the ratio of 18:3n6 to 18:2n6 )	3.1 (2.9–3.4)	3.0 (2.8–3.2)	2.9 (2.7–3.1)	3.2 (2.9–3.3)	3.3 (3.0–3.5)	3.2 (3.0–3.4)	0.127
Δ5 desaturase (the ratio of 20:4n6 to 20:3n6 )	0.009 (0.008–0.01)	0.009 (0.007–0.01)	0.009 (0.007–0.01)	0.007 (0.005–0.009)	0.005 (0.003–0.006)	0.007 (0.005–0.009)	0.161
Δ9 desaturase (the ratio of 16:1n7 to 16:00 )	0.52 (0.51–0.53)	0.52 (0.51–0.52)	0.55 (0.54–0.56)**	0.51 (0.5–0.52)	0.5 (0.49–0.51)	0.52 (0.51–0.53)*	0.14

Data are means±95% confidence intervals. Listed *P*-values are from analysis of variance (ANOVA) for interaction between group (vegetarian and control group) and time (0,12 and 24 weeks). Significant changes from baseline to 12 weeks and from 12 to 24 weeks are indicated by * for *P*<0.05, ** for *P*<0.01 and *** for *P*<0.001.
